# The ONLINE-TICS Study Protocol: A Randomized Observer-Blind Clinical Trial to Demonstrate the Efficacy and Safety of Internet-Delivered Behavioral Treatment for Adults with Chronic Tic Disorders

**DOI:** 10.3389/fpsyt.2016.00119

**Published:** 2016-06-30

**Authors:** Ewgeni Jakubovski, Cornelia Reichert, Annika Karch, Nadine Buddensiek, Daniel Breuer, Kirsten Müller-Vahl

**Affiliations:** ^1^Department of Psychiatry, Socialpsychiatry and Psychotherapy, Hannover Medical School, Hannover, Germany; ^2^Institute for Biostatistics, Hannover Medical School, Hannover, Germany; ^3^Clinical Trial Center, Hannover Medical School, Hannover, Germany

**Keywords:** Tourette syndrome, tics, comprehensive behavioral intervention for tics, Internet-delivered comprehensive behavioral intervention for tics, habit reversal training, tele-health program

## Abstract

**Background:**

In recent years, behavioral therapy with comprehensive behavioral intervention for tics (CBIT) has been recognized as an effective and safe treatment in patients with Gilles de la Tourette syndrome. In Germany, however, dissemination of CBIT is restricted due to a considerable lack of well-trained therapists. The aim of this study is to overcome this deficiency by creating a new and sophisticated Internet-delivered CBIT (iCBIT) program. With this study, we want to demonstrate that iCBIT is superior to Internet-delivered psychoeducation and comparable to face-to-face CBIT.

**Method and analysis:**

This is a multicenter, prospective, randomized, controlled, observer-blind clinical trial, which will be conducted at five sites in Germany (ONLINE-TICS). Over the course of 2 years, 160 adult patients with chronic tic disorders will be assigned to one of three treatment arms: iCBIT (*n* = 72), online psychoeducation (*n* = 72), or face-to-face CBIT (*n* = 16). All treatments will consist of eighty therapy sessions over a period of 10 weeks and will follow the well-established CBIT manual by Woods and colleagues. The primary outcome measure will be the change in Yale Global Tic Severity Scale (YGTSS) at 1-week posttreatment. Secondary outcome measures include YGTSS change at 3 and 6 months, video- and self-ratings of tics as well as scales for psychiatric comorbidities assessed at each visit. The primary analysis will compare iCBIT to online psychoeducation using a mixed linear model with the YGTSS change as dependent variable. Secondary analyses will look at the comparison between iCBIT and face-to-face CBIT in a non-inferiority analysis.

**Discussion:**

If iCBIT proves to be effective, it would be a considerable contribution to close the wide gap in treatment providers for tic disorders not only in Germany but also in several other countries, since this Internet-delivered therapy does not require the supervision of a therapist. In addition, iCBIT would be a cost-effective and readily available treatment alternative that guarantees high quality standard of CBIT.

**Ethics and dissemination:**

All institutional review boards approve the protocol. All participants will provide informed consent. There are no conflicts of interest. After study completion, the results will be published.

**Study registration:**

ClinicalTrials.gov Identifier: NCT02413216.

## Introduction

Gilles de la Tourette syndrome (TS) is a chronic neuropsychiatric disorder of childhood onset characterized by multiple motor and vocal tics. While tics are described and diagnosed phenotypically, there is pathophysiological evidence for an involvement of cortico-striato-thalamo-cortical circuits. Tic disorders are common with a prevalence of 0.4–3.8% (1). The majority of patients additionally suffers from comorbidities such as attention deficit hyperactivity disorder (ADHD), obsessive-compulsive disorder (OCD), depression, anxiety, and self-injurious behavior (2). Quality of life is significantly impaired in a substantial number of patients not only due to tics and comorbidities but also because of ignorance and a lack of information, leading to bullying and stigmatization. Even today, it takes on average more than 5 years to make the correct diagnosis (3). According to a recent German study, TS is a cost-intensive disease that causes high direct and in particular high indirect costs (average total annual disease specific costs in Germany = €3404) including indirect medical costs for productivity loss of €2511 ± 3810 and for absenteeism of €260 ± 1184 (4). Thus, TS poses a considerable burden not only to patients but also to health-care providers.

For many years, dopamine receptor antagonists have been recommended as first choice treatment for tics, although these drugs are often associated with significant adverse effects. In Germany and several other countries, however, only haloperidol is officially licensed for the treatment of TS (5). Therefore, a large number of other substances (including clonidine, tetrabenazine, dopamine agonists, botulinum toxin, and cannabinoids) has been suggested for the treatment of tics. However, for most of these medications evidence is limited.

Data of two large recently conducted and well-powered randomized controlled trials (RCT) – including more than 100 patients each – confirmed preliminary results and demonstrated that behavioral therapy with Comprehensive Behavioral Intervention for Tics (CBIT) is an effective and safe treatment for managing the tics of children and adults with TS (6, 7). On average, CBIT treatment results in a tic improvement of about 30% [for review, see Dutta and Cavanna (8) and Verdellen et al. (9)]. CBIT is a short-term behavioral therapy consisting of only eight sessions over 10 weeks that includes psychoeducation, habit reversal training (HRT) with competing-response training (CRT), functional analysis, and relaxation training (progressive muscle relaxation). The treatment benefit of CBIT was even shown to persist over a period of 3 and 6 months (6, 7). Its effectiveness in conjunction with the lack of side effects are clear benefits of CBIT, and are advantages over currently used medications, offering thus a competitive alternative for treating tic disorders. Accordingly, in the latest guidelines – including those of the European Society for the Study of Tourette Syndrome (ESSTS) (5) – behavioral therapy with either CBIT or Exposure and Response Prevention Training (ERP) (9) is now recommended as the first-line treatment for tic disorders (10).

However in Germany and most other European countries, this recommendation cannot be implemented, since there is a considerable lack of psychotherapists trained in CBIT. Motivated by these significant barriers to dissemination, a recent study compared the effect of face-to-face to video-delivered CBIT demonstrating comparable efficacy and acceptability (11). However, while video-delivered CBIT – compared to face-to-face CBIT – is independent of the location, it is still dependent on the availability of well-trained therapists conducting the video sessions. Unfortunately, this is not the case in many European countries, including Germany, where therapists trained and experienced in CBIT are lacking on a national scale. As of today, there is even no therapy manual or patient workbook available for the treatment of adult patients with TS in German language.

In other psychiatric conditions such as depression (12, 13) and anxiety disorders (14), Internet-delivered self-guided psychotherapy (using cognitive behavioral therapy, interpersonal psychotherapy and psychodynamic treatment) has been shown to be superior to a control condition and comparably effective as face-to-face psychotherapy (15). Due to the simple nature of the exercises involved in CBIT, we have reason to assume that CBIT is particularly well suited to be delivered *via* Internet compared to those more complex psychotherapeutic interventions. Accordingly, in a recently published meta-analysis on behavior therapy for TS, the authors stated that “a major barrier to wider implementation of CBIT and HRT is that few therapists are trained in their use” and concluded that “broader distribution of behavior therapy through increased training or tele-health methods is encouraged” (16).

Therefore, the aim of this study will be to develop and test a fully self-sufficient Internet-delivered CBIT (iCBIT) for adult patients with TS, with no therapist involved in any way (ONLINE-TICS). Although interventions *via* video, Skype, or smartphone have been suggested (11, 17, 18) and, in addition, an RCT started only recently testing the efficacy of a computerized, self-administered version of CBIT (called http://TicHelper.com) in 64 children and adolescents with tics (age 8–18 years) (http://ClinicalTrials.gov Identifier: NCT02413216), to the best of our knowledge, so far, there is no iCBIT program available in any language for the treatment of tics in adult patients with TS and other chronic tic disorders. Since in TS – due to the natural waxing and waning course of tics – it is difficult to demonstrate efficacy of a treatment, only a well-designed and sufficiently powered study is suitable to demonstrate efficacy. Therefore, our study will be a multicenter, prospective, controlled, randomized, observer-blind clinical trial that aims to include 160 patients. ONLINE-TICS will be funded by the Federal Ministry of Education and Research (BMBF) in Germany (BMBF: 01KG1421). It is designed to examine the efficacy of an iCBIT intervention as compared to (1) a placebo platform consisting of psychoeducation only – which is our primary analysis – and (2) a conventional face-to-face CBIT intervention – which is our secondary analysis. We hypothesize that iCBIT (1) is superior to the placebo platform and (2) has a comparable effect size to the face-to-face treatment arm. Planned participating sites are Hannover Medical School (MHH), University of Munich, University of Aachen, University of Lübeck, and University of Dresden.

## Methods and Analysis

### Study Sample

Over the course of 2 years, 160 patients will be enrolled in this study. The recruitment will run primarily through the study centers’ outpatient clinics. Moreover, further advertisement will be carried out by German self-help and advocacy groups, newsletters, and annual meetings. Patients who are interested in participation will be referred to the study centers, where they will be informed about the details of the study and an appointment for a screening visit will be made.

During screening, patients will be informed (orally and in writing) about clinical assessments and randomized allocation. Those patients who will not be randomized to iCBIT during the RCT, will have the opportunity of receiving iCBIT after study completion (i.e., after their follow-up assessments). There will be no financial compensation for the study participation. However, travel costs will be reimbursed. Before being enrolled, patients will have to provide written consent. We expect a screening rate of 280 patients in 2 years, out of which about 240 patients are expected to meet the inclusion criteria for this study, out of which 160 should be willing to participate and will be included. Planned recruitment by study site is displayed on Table 1.

**Table 1:** **Numbers of patients approximately recruited per center**.

	Center	Principal investigator	Expected *n* of patients recruited for the complete trial
1	Hannover Medical School	Müller-Vahl	62
2	Ludwig-Maximilians-University Munich	Müller	28
3	University of Lübeck	Münchau	24
4	University Hospital Aachen	Neuner	26
5	University of Dresden	Roessner	20
	Total		160

*n, number*.

### Study Design

This is a multicenter, prospective, randomized, controlled, observer-blind clinical trial on the efficacy of iCBIT in the treatment of tics in adult patients with TS or other chronic tic disorders. For four of the five planned study sites (except for Hanover), a two-armed study design will be used – consisting of a placebo-treatment arm (Internet-based psychoeducation) and an iCBIT-treatment arm. Only in Hannover (MHH), an additional face-to-face CBIT treatment arm will be added. Hannover is the only center offering the face-to-face treatment due to the lack of well-trained therapists in Germany even in centers specializing in TS.

The time from first patient in to last patient out is expected to be 33 months consisting of the recruitment phase (24 months) plus the treatment phase (10 weeks) plus a follow-up (6 months). The drop-out rate is expected to be less than 10%.

This study is registered on ClinicalTrials.gov Identifier: NCT02605902.

### Eligibility Criteria

The following inclusion criteria were defined:

– chronic tic disorder or TS according to DSM-5
– age ≥18 years
– total tic score of the Yale Global Tic Severity Scale (YGTSS–TTS) >14 (for patients with TS and YGTSS–TTS >10 for patients with other chronic tic disorders)
– CGI-S >4 at baseline
– If patients receive additional medical or surgical treatment for tics and psychiatric comorbidities, they must be on a stable dose for at least 6 weeks before entering the study
– fluency in German language
– written informed consent.

Exclusion criteria for this study are:

– history of schizophrenia or pervasive developmental disorders
– current diagnosis of substance abuse or dependence
– primary comorbidities such as OCD, ADHD, depression, anxiety disorder, in need of therapy
– history of behavioral treatment for tics (CBIT, HRT, ERP)
– secondary tic disorders and other significant neurological and psychiatric diseases (such as schizophrenia, thought disorder)
– no access to Internet or inability to operate a computer
– participation in any interventional clinical study investigating drugs/medical devices 6 weeks prior to study enrollment.

These broad inclusion and exclusion criteria were based on the study by Wilhelm et al. (7), and guarantee that a representative group of TS treatment-seeking patients will be included, in particular, patients (1) with moderate or severe tics, (2) with simple or complex tics, (3) with or without comorbidities, (4) with or without additional drug treatment, (5) with short or long disease duration, and (6) of different age groups.

### Randomization

Potential bias will be minimized by randomized treatment allocation. The randomization list will be based on permuted blocks and is stratified by study site and pre-existing medication for the treatment of tics (yes/no). There will be a 1:1 randomization in four/five centers (for reasons explained above), and only in Hannover patients will be randomized 1:1:1 until 16 patients are randomized to face-to-face CBIT and 1:1 afterward. The randomization will be conducted centrally *via* web randomization. Patients will be given a sealed envelope by the investigator containing the access code for the therapy platform (or – only at the MHH center – information for face-to-face CBIT).

### Blinding

The study is observer blind. Although the patients will receive no information as to which treatment arm they are assigned to, it can be assumed that patients will find out. This will definitely be the case in the face-to-face CBIT treatment arm, but will most likely happen in the iCBIT – and placebo treatment arms as well, since all patients will be informed about the study objectives and the contents of the possible treatment arms. Thus, a genuine blinding of patients will not be possible. This represents a fundamental problem in all studies with psychotherapeutic interventions. However, much effort is being put into avoiding the unblinding of study physicians:

- The examiners involved in the study will only carry out assessments. Apart from this, they will not be responsible for the treatment of patients during the study period and will not answer questions related to the therapy.
- Patients will be instructed in writing, neither to talk nor to ask questions about the contents of the therapy during the study visits. In addition, patients will be explicitly asked by the examiner at the beginning of each visit (telephone and personal study visits), not to talk about the contents of their therapy. Technical as well as content-related assistance related to the online platforms will be provided *via* a central hotline located in Hanover through a study staff member who will not be involved in assessments.
- Despite these precautions, a blinding of the evaluating study physicians cannot be guaranteed to 100%. If an investigator will be unblinded by a patient, this will be documented directly at the respective visit. Whenever possible, the patient will be reassessed by an alternative unblinded investigator at this visit (if unblinding happened before the completion of this study visit) and at all following visits.
- The therapist, who will treat patients with face-to-face CBIT within this study will not be involved in clinical assessments.
- The recording and scoring of videos for tic assessment will not take place in the same center (videos will be analyzed centrally at the MHH) and only after termination of the study, thus blinding of the evaluating physician is guaranteed.

### Compliance

In order to ensure that the patients will take active part in the Internet-based intervention, several automated checkups are integrated into the online platform for two purposes: first, it allows to organize and motivate the patients with regard to their sessions (missed sessions, time in session, active reminder, and more). Second, a direct quantification of the compliance is possible after study completion and compliance can be analyzed statistically.

To all patients randomized to the placebo platform, an (open, uncontrolled) participation in the iCBIT therapy will be offered after study completion. However, this will only be possible for those patients in the face-to-face CBIT and the placebo treatment arms, who have been compliant before and completed the randomized treatment.

### Internet-Delivered CBIT

Comprehensive Behavioral Intervention for Tics can be regarded as an “extension” of HRT developed for the treatment of patients with tics including psychoeducation, tic-awareness training (including the awareness of premonitory urges preceding the occurrence of the tics), CRT, relaxation training, and functional analysis to identify events and situations that influence tic severity in order to develop coping strategies to manage these situations. HRT, the primary component of CBIT, is based on the premise that tics are maintained by response chaining, lack of awareness of their occurrence, excessive practice, social reinforcement, and tolerance of the tics (19). The core element of HRT, in turn, is CRT that aims to introduce a voluntarily performed non-tic movement that is physically incompatible with the performance of the tic. Thus, the competing response encourages the subject to respond to the urge by performing a movement competing with the tic. Over time, performance of the competing response breaks the cycle between the premonitory urge and the relief following the tic.

The iCBIT online platform is being created by the authors (Kirsten Müller-Vahl, Nadine Buddensiek, Ewgeni Jakubovski, and Cornelia Reichert) and in particular by Kirsten Müller-Vahl, who is well experienced in all aspects of TS, and Nadine Buddensiek, who is an experienced and well-trained psychotherapist for CBIT in adults. It is following the manual for face-to-face CBIT by Woods (20) with respect to both, number and content of treatment sessions, distribution of CBIT elements to particular treatment sessions, and duration of treatment. Thus, the only difference between this conventional and well established form of CBIT and iCBIT will be the route of delivery, on the one hand *via* face-to-face and on the other hand *via* Internet. Only very few adaptations have been inevitable due to Internet-delivery such as more extensive psychoeducation, description of CBIT at great length and inclusion of additional contents. For example, for better illustration, videos will be included where patients as well as an expert clinician (KMV) talk about their own experience with both CBIT and TS as well as general aspects of TS and contents of CBIT. Additional content will be offered in form of a FAQ section, which is meant to provide answers to most frequent questions that might arise over the course of the program. Comparable to the Woods’ manual for face-to-face CBIT, other resources are provided including information about the TS, working materials such as “Personal Tic Sheet,” “The Tic Symptom Hierarchy Tracker,” “Functional Assessment Form” (to help patients identify situations that deteriorate their tics in order to develop specific function-based interventions), “Tic Hassle Form,” a list of possible competing responses for different tics, and a reward program. Working sheets can either be used online or can be printed out for use in paper form. A part of the working materials can alternatively be used *via* smartphone app.

In line with the CBIT manual provided by Woods (20), iCBIT will consist of eight sessions of which the first two session will take 90 min, while the other six sessions will take 60 min. The first six sessions will be weekly and the last two sessions will be biweekly, totaling to an overall treatment duration of 10 weeks. However, iCBIT will allow patients more time flexibility than conventional face-to-face CBIT.

### Placebo: Online Psychoeducation

The control group will consist of psychoeducation only. It will match the iCBIT group in terms of the number and duration of sessions (eight Internet-delivered sessions over a period of 10 weeks). Patients in the control group will receive no elements of CBIT. Psychoeducation will include additional disorder-specific information. A comparable design has been chosen in available large RCTs using face-to-face CBIT (6, 7). Due to the well-known disadvantages of waiting list designs, we decided against using it in our study (21).

### Face-to-Face CBIT

This additional treatment arm will only be offered in Hanover. The face-to-face CBIT therapy will follow the CBIT manual by Woods et al. (22). In addition, each face-to-face session will correspond exactly to the iCBIT sessions: treatment will thus consist of eight sessions of which the first two sessions will last 90 min and the remaining six sessions 60 min (total of 10 weeks).

### Booster Sessions

In the period of time from the end of active treatment until the last follow-up visit (see below), patients in all treatment arms will get the option of receiving booster sessions to refresh the therapy. There will be no limitation in terms of number, scope, and duration of these additional sessions in the Internet-based treatment arms. In these sessions, patients will have access to information earlier provided in the eight sessions of their treatment module. However, no new information or contents will be offered. In the face-to-face CBIT group, up to two booster sessions will be possible in terms of face-to-face CBIT sessions with the therapist [comparable to the CBIT manual by Woods et al. (22) and recent studies (7)]. The duration of these sessions will be 60 min each.

### Online Platform: Minddistrict

The technical implementation of the Internet-based treatment is being set in place in cooperation with the Minddistrict company. All treatment-related contents for the online modules are being developed by the research team at the MHH. In the final stage of development, the platform will be reviewed by the authors of the original CBIT manual Douglas Woods and Sabine Wilhelm (who is a German native speaker). Prior to study begin, the platform will be additionally pilot tested by a small group of patients with tic disorders to verify its practicability and usability. Feedback will be used for further optimization of the platform before study launch.

### Outcome Measures

The primary outcome measure will be the YGTSS–TTS (23) 1 week after end of treatment. This has also been used in studies demonstrating efficacy of face-to-face CBIT (6, 7). Tic severity will further be assessed *via* several secondary outcome measurements using (i) the YGTSS–TTS 3 and 6 months after end of treatment, (ii) the Modified Rush Video-Based Tic Rating Scale (MRVS) (24), and (iii) the Adult Tic Questionnaire (ATQ), a tic self-rating scale, which is parallel in format and content to the Parent Tic Questionnaire (25).

The following further secondary outcome measures will be included: the Premonitory Urge of Tics Scale (PUTS) (26), Beck Depression Inventory (BDI-II) (27), Conners’ Adult ADHD Rating Scale (CAARS) (28), Yale-Brown Obsessive-Compulsive Scale (Y-BOCS) (29, 30), Beck Anxiety Inventory (BAI) (31), Gilles de la Tourette Syndrome-Quality of Life Scale (GTS-QoL) (32). In addition, the Clinical Global Impression – Severity Score (CGI-S) and the Clinical Global Impression – Improvement Score (CGI-I) will be used to measure overall severity of disease and improvement at follow-up. An adopted version of the Working Alliance Inventory-Short Revised (WAI-SR) will be applied to assess the therapeutic alliance (33).

### Schedule of Assessments

All clinical assessments will be performed at screening, baseline, week 5 (during treatment), week 11 (1 week after end of treatment), and 3 and 6 months after end of treatment. Follow-up assessments at 3 and 6 months after the end of treatment will provide estimates of the durability of treatment effect. Thus, time points of assessment in this study are comparable to those in the study by Wilhelm et al. (7), which recently demonstrated that face-to-face CBIT is an effective and safe intervention in adults with TS. Randomization will be performed at the baseline visit. Screening and baseline visits should not be more than 4 weeks apart, otherwise the patient will have to be rescreened. A detailed assessment schedule is provided on Table 2. At week 17 and 29 (1.5 and 4.5 months after the end of treatment), telephone visits will take place. These additional visits serve the purpose of improving compliance in order to keep the drop-out rate as low as possible. During these telephone visits there will be no further testing.

**Table 2:** **Assessment schedule**.

Study period	Screening	Baseline	Treatment: (a) iCBIT, (b) placebo, (c) Face-to-face CBIT								Follow-up visits/booster-treatment^a^				
**Visit**	1	1					2				3		4		5
**Week**	−4 −0	0	1	2	3	4	5	6	8	10	11	17	23	29	35
**Treatment-session**			1	2	3	4	5	6	7	8	Frequency individual, for face-to-face CBIT: max = 2				
**General procedures**															
Written informed consent	X														
Inclusion/exclusion criteria	X														
Demographics	X														
Medical and medication history	X														
**Intervention**															
Randomization		X													
Compliance^b^															
– Asking for compliance			X	X	X	X	X	X	X	X	X	X	X	X	X
– Phone visits												X		X	
– Online check-ups			X	X	X	X	X	X	X	X					
**Psychometric assessment**															
Tics:															
– YGTSS	X	X					X				X		X		X
– ATQ		X					X				X		X		X
– MRVS		X					X				X		X		X
Severity of disease: CGI-S	X	X					X				X		X		X
Improvement of disease: CGI-I							X				X		X		X
Premonitory urges: PUTS		X					X				X		X		X
Quality of life: GTS-QoL		X					X				X		X		X
Mood: BDI-II		X					X				X		X		X
Anxiety: BAI		X					X				X		X		X
ADHD:															
– DSM-IV symptom list		X													
– CAARS		X					X				X		X		X
OCD: Y-BOCS		X					X				X		X		X
**Adverse events**															
Open question							X				X		X		X
**Therapeutic alliance**															
WAI-SR							X				X		X		X

*YGTSS, Yale Global Tic Severity Scale; MRVS, Modified Rush Video-Based Tic Rating Scale; ATQ, Adult Tic Questionnaire; CGI-S, Clinical Global Impression–Severity Score; CGI-I, Clinical Global Impression-Improvement Score; PUTS, Premonitory Urge of Tics Scale; GTS-QoL, Gilles de la Tourette Syndrome-Quality of Life Scale; BDI-II, Beck Depression Inventory; ADHD, Attention Deficit Hyperactivity Disorder; CAARS, Conner’s Adult ADHD Rating Scale; OCD, Obsessive-Compulsive Disorder; Y-BOCS, Yale-Brown Obsessive-Compulsive Scale; BAI, Beck Anxiety Inventory, WAI-SR, Working Alliance Inventory-Short Revised*.

*^a^Booster-sessions: booster-session are optional. Frequency, scope, and number will vary individually in the iCBIT and placebo groups, in the face-to -face CBIT group up to two booster sessions a 60-min between week 11 and 35 are possible*.

*^b^Compliance: the compliance of patients in all treatment arms will be assessed during the telephone and in person visits. In the face-to-face CBIT treatment, arm compliance will also be determined via regular and full participation in the therapy sessions. In the iCBIT and placebo groups, the participation in the therapy session will be determined automatically by the online platform by collecting information on when, how long and where the patients were logged in*.

### Sample Size Calculation

The sample size calculation is based on two studies in which the efficacy of face-to-face CBIT was compared with face-to-face psychoeducation in adult and pediatric patients with TS and chronic tic disorders (6, 7). The average YGTSS–TTS improvement across both studies was 3.5 (±5.5 SD), when comparing face-to-face CBIT to psychoeducation. The basic assumption of our study is that iCBIT is as effective as face-to-face CBIT. However, we expect a mean difference of 3.0 instead of 3.5 for the sample size estimation since only adult patients will be enrolled in this study, and a difference of three points is considered relevant enough to be detectable.

Under these conditions and with a one-sided type I error of 2.5%, *n* = 72 patients per treatment arm are required to reach a power of 90%. Therefore, a total of 144 patients will be included in the Internet-based treatment arms iCBIT and placebo. The expected drop-out rate is considered very low (<10%) based on previous studies in our centers and the studies by Wilhelm et al. (7) and Piacentini et al. (6). But even with as low as 60–65 patients per treatment arm a power of 84–86% can be achieved.

Sample size for the face-to-face CBIT arm in Hannover is feasibility driven. Sixteen patients will be randomized to face-to-face CBIT. The study is not powered for this secondary non-inferiority analysis.

### Data Analysis

The primary outcome measure (YGTSS–TTS) will be used as a continuous variable in the primary statistical analysis. In secondary analyses, YGTSS–TTS and CGI-I will be used as binary covariates for responder analysis: response is defined by a 30% decrease (YGTSS–TTS) and an improvement of 1–2 = much or very much improved (CGI-I), respectively.

#### Primary Analysis

The primary analysis will be performed in the intention-to-treat population. A mixed linear model will be used with change in YGTSS–TTS score (follow-up minus baseline) as the outcome variable. Therapy (iCBIT versus placebo), study site, concomitant tics medications (yes versus no), YGTSS–TTS baseline score, and YGTSS-TTS assessment time point (baseline, week 5, and 1 week after end of treatment) as well as an interaction of therapy and time point will be included as fixed effects in the model. The patient variable will be considered a random effect. The mixed model will be analyzed *via* PROC MIXED procedure in SAS statistical software. The therapeutic effect (iCBIT minus placebo) at week 1 after the end of treatment and the associated two-sided 95% confidence interval will be obtained from this analysis. Superiority of iCBIT will be shown if the upper limit of this confidence interval is less than 0.

Various sensitivity analyses are planned (e.g., in a per-protocol population, using analysis of covariance and more). In the unlikely event that the mixed model will not converge in the primary analysis, an analysis of covariance with last observation carried forward will be carried out instead.

#### Secondary Analyses

The key secondary analysis is the comparison of iCBIT to face-to-face CBIT in terms of YGTSS–TTS change 1-week posttreatment. If the primary objective of superiority of iCBIT over Placebo can be shown, this key secondary non-inferiority hypothesis can be tested in a confirmatory analysis with a one-sided significance level of 2.5%. The non-inferiority margin for the mean difference (iCBIT versus face-to-face CBIT) will be set to 3. The statistical analysis model is in line with the primary analysis described above (mixed model).

In further secondary analyses, the mean change from baseline to each time point will be tested in MRVS, ATQ, PUTS, GTS-QoL, WAI-SR, and the psychiatric comorbidity scores by means of an analysis of covariance between the treatment groups. The analyses will be adjusted for study site and concomitant tic medications as well as the baseline score of the respective outcome variable. Several subgroup analyses are planned as a function of pre-stratification-variables: comorbidities, age, gender, and duration of disorder. Further analyses investigating interactions terms will be conducted in order to assess the robustness of the treatment effect across the strata. Proportion of responders will be analyzed with logistic regression stratified in line with the primary analysis. All secondary endpoints will be analyzed in an intention-to-treat population.

### Quality Assurance and Monitoring

To assure data quality and patients’ safety, regular monitoring visits will be carried out. An on-site initiation visit has to be performed, before a site is allowed to start recruitment. Periodic monitoring visits and source data verification will be done according to a risk-adapted approach (34). Close out visits will be done at the end of the trial and in case a site will be closed. Project managers and monitors will stay in close and regular contact with all trial sites.

Quality assurance will be realized by in-house monitoring *via* electronic data capture. On site source data verification will be done 100% for the first patient at each center and 20–50% reduced risk adapted monitoring afterward. In total, 16 periodic monitoring visits are planned (on average, two to three visits/site). If applicable, monitoring visits will be adapted according to recruitment.

### Safety

For this study, no safety issues have been identified. Adverse events and incidents will be documented *via* open questions and will be analyzed descriptively. Patients can contact the recruiting center at any time. If required, however, unblinding will be possible at any time.

## Discussion

### Clinical Implications

This is the first study examining the efficacy and safety of a fully self-sufficient Internet-delivered behavioral therapy using CBIT for adult patients with chronic tic disorders that does not require the supervision of a therapist (ONLINE-TICS). We will carry out a large multicenter, RCT in five different German TS centers to compare the efficacy of iCBIT [an online therapy, which follows closely the CBIT manual by Woods (20)] to (1) an online placebo – containing only psychoeducation – and to (2) a conventional and well-established face-to-face CBIT treatment. The inclusion of both an online-delivered placebo intervention and face-to-face CBIT gives us the unique possibility to investigate not only the superiority of iCBIT over placebo but also the non-inferiority compared to face-to-face CBIT.

We expect iCBIT to outperform the psychoeducation online platform (placebo) and to show a treatment effect comparable to face-to-face therapy. If shown to be effective, iCBIT will have several major advantages compared to face-to-face CBIT: (i) iCBIT can be delivered to any patient (the only requirement is a computer with Internet access), (ii) treatment of patients with TS according to latest guidelines will no longer be hampered by the lack of therapists trained in CBIT, (iii) iCBIT will shorten waiting time, (iv) iCBIT may be a treatment option even for those patients who refuse face-to-face psychotherapy due to reasons such as effort, costs, difficulties in reaching the therapist’s office (for example because of significant tics or a comorbid anxiety disorder), and personal career (since for example in Germany an appointment as a tenured German civil servant is no longer possible after a person has submitted an application for psychotherapy to his statutory health insurance), (v) costs for iCBIT will be much lower compared to costs for face-to-face CBIT, (vi) iCBIT guarantees highest quality standards, and (vii) there is evidence that Internet-delivered therapy in general reaches other groups of patients (e.g., homemakers, higher-educated people, employees, elderly people) as compared to regular face-to-face therapy (35). Therefore, we can assume that iCBIT will be an effective, safe, and cost-effective treatment for a substantial number of patients with TS. Thus, if effective, iCBIT will bridge a worldwide healthcare gap. In addition, one could think about combining elements of both types of treatment, face-to-face CBIT and iCBIT in order to improve efficacy (by giving the patient the possibility for timely flexible repetitions and the use of additional content such as videos, FAQ, and detailed psychoeducation) and flexibility (by using alternatively two different routes of delivery for CBIT) and as an aid for therapists in their work routine.

### Limitations

All of the treatment arms in our study compare behavioral interventions, but there is no comparison to medical treatment. Due to several reasons, we decided not to use a pharmacotherapy as an active comparator: (i) in Germany, only haloperidol is licensed for the treatment of tics, but it is no longer recommended due to significant adverse effects (5), (ii) due to lack of controlled clinical trials, the efficacy of those drugs most often used at least in Germany (tiapride, risperidone, and aripiprazole) is unknown (5) and, therefore, these drugs cannot be used as active comparators, (iii) until today there is no trial available comparing directly the efficacy of behavioral therapy versus pharmacotherapy, and (iv) using one of those drugs most often used for the treatment of tics would limit patient population, since a substantial number of patients would refuse participation.

Our study cannot be considered double-blind, since it will be very easy for patients to figure out if they are receiving iCBIT or not. This is a common problem that most studies examining psychotherapeutic interventions face. Nevertheless, our study has a high external validity. Treatment will not take place in a clinical setting, but in the homes of the participants. All treatment-related exercises will take place in patients’ everyday lives. There will be no issue with dissemination, since once shown to be effective, iCBIT can be made accessible to anybody seeking treatment for tics and will be delivered in the very same way, which was shown to be effective. We decided not to use a waiting list control designs, since it is well known that these trials may overestimate treatment effects. In order to make the control group more attractive, patients randomized to the control group will receive interesting and helpful information about their disorder in an entertaining presentation and, in addition, will have the possibility to receive iCBIT after study completion.

Our study examines an online treatment that works completely without the involvement of a therapist. Does that make therapists obsolete? Our answer is: definitely not. In our opinion, iCBIT offers a solution to overcome the lack of well-trained therapists. However, we do not intend to propose a program to replace consultation with a qualified medical doctor. An online treatment like ours cannot diagnose tics or test the indication for treatment; this always has to be done by a mental health specialist. Oftentimes, a patient might have several indications for treatment of which treatment for tics might be a minor one. This diagnostic work always has to be done by an expert. That being said, our platform will be a very useful tool to supplement therapeutic work in the private practice. The platform could assist the patient with regular homework and exercises, while a therapist could focus on helping the patient troubleshoot as well as work on other issues that the patient might have. This would be a timesaving combination for both the patient and the therapist.

### Conclusion

Our study ONLINE-TICS will test the efficacy of iCBIT – an online version of the highly effective face-to-face CBIT, which is the current first-line treatment for tic disorder. If shown to be effective, it will have the potential to bridge a large gap in the current health-care system in the treatment of tic disorders in Germany.

## Ethics and Dissemination

### Informed Consent and Institutional Review Boards

The study is based on the principles of Good Clinical Practice (GCP), according to the Declaration of Helsinki. Patients will be given oral and written explanation of the study including its potential risks, their right to withdraw consent at any time and the details of data protection and confidentiality and sufficient time to ask questions. A signed consent form will be obtained. The patient information and a copy of the signed consent form will be handed to the patient. The data will be monitored by HCTC. All documents and information will be treated with strict confidentiality.

Each study site will only be able to start data collection once the local Institutional Review Boards (IRB) approval is obtained. In the case of protocol changes an amendment will be submitted to the concerning IRB.

### Confidentiality

The information collected in the study, especially the information related to the identity of the patient, will be confidential and protected by law. The data collected at each study site will be stored and analyzed in de-identified form and kept for a period of 10 years in a lockable cabinet or password protected computer.

The collected data will be only accessible to the principal investigator and study staff of the respective study site as well as the monitors.

The Minddistrict company will not have access to any personal data of the study participants. The necessary login data will be provided and managed by the MHH research personal. Additionally, the Minddistrict portal will be SSL-encrypted.

Video recordings from all participating study sites will be saved on an encrypted cloud offered by the University of Goettingen.

### Dissemination

After study completion, the results of the primary and secondary analyses will be published in international peer-reviewed journals.

If shown to be effective the therapy platform will be made available to the general public in Germany in an appropriate manner. For this purpose, all necessary contractual arrangements between the MHH and the company Minddistrict will be clarified and defined before the beginning of the study. Currently, all rights to the Internet platform iCBIT belong to the MHH represented by KMV.

## Author Contributions

EJ: manuscript preparation and correspondance, assistance in generating therapy platform, later involvement as a study investigator. CR: assistance in manuscript preparation, study site coordination, planning of study procedures, later involvement as a study investigator. AK: study statistician, assistance in manuscript preparation, methodological consultant. NB: assistance in generating therapy platform, later study therapist for treatment arm. DB: study monitor, ensures compliance with GCP throughout the study. KM-V: principal investigator, involved in all study and writing-related tasks as major supervisor. All authors approved of the final version of the manuscript.

## Conflict of Interest Statement

The authors declare that the research was conducted in the absence of any commercial or financial relationships that could be construed as a potential conflict of interest.
